# AIDS-Kaposi Sarcoma and Classic Kaposi Sarcoma: are different ultrasound patterns related to different variants?

**DOI:** 10.1186/1756-9966-30-40

**Published:** 2011-04-13

**Authors:** Francesco M Solivetti, Fulvia Elia, Alessandra Latini, Carlo Cota, Paola Cordiali-Fei, Aldo Di Carlo

**Affiliations:** 1 Radiology Department, San Gallicano Dermatology Institute - Rome - Italy; 2 Infective Dermatology Division, San Gallicano Dermatology Institute - Rome - Italy; 3 Dermatopathology Division, San Gallicano Dermatology Institute - Rome - Italy; 4 Clinical Pathology and Microbiology Division, San Gallicano Dermatology Institute - Rome - Italy; 5 Scientific Director, San Gallicano Dermatology Institute - Rome - Italy

## Abstract

**Background:**

Kaposi Sarcoma (KS) is a malignancy of endothelial skin cells with multifocal localization on the skin, lymph nodes and visceral organs. Although all clinical variants are associated with HHV-8 infection, specific differences in the clinical onset and in the natural history of AIDS-KS and Classic-KS have been described. The present randomised prospective-observational study aimed to investigate whether the ultrasound pattern and color Doppler flow imaging of vascularisation of skin lesions of patients with Classic KS (CKS) or AIDS-KS could provide useful information to the evaluation of clinical activity of the disease.

**Methods:**

Cutaneous lesions of 24 patients with histologically confirmed KS were investigated using very high frequency ultrasound probes; 16 patients had CKS and 8 had AIDS-KS. HHV-8 infection was confirmed in all patients by investigating the specific humoral response to viral antigens. Immunological and virological parameters were also assessed to monitor HIV or HHV-8 viral infection. For each patient, a target skin lesion was selected on the basis of size (diameter from 0.4 to 2 cm). Each lesion was analyzed in terms of size, depth and color Doppler pattern.

**Results:**

The B-mode ultrasound patterns of skin lesions did not differ when comparing CKS patients to AIDS-KS patients, whereas the color Doppler signal, which is associated with vascular activity, was detected in the KS lesions of 6/8 AIDS-KS patients (75.0%) and in 2/16 CKS (16,7%); the latter two patients showed a clinically progressive and extensive disease stage (IV B).

**Conclusions:**

Our preliminary results suggest that small cutaneous KS lesions - in both CKS and AIDS-KS patients- display similar B-mode ultrasound patterns ( hypoechoic, well defined, superficial lesions). However, the color Doppler signal, which is associated with endothelial activity and angiogenesis, which play a substantial role in KS progression, could constitute a useful tool for evaluating disease activity.

## Background

Kaposi's Sarcoma (KS) is a tumour affecting mainly the skin, with multifocal expression and possible lymph nodal and visceral involvement [1]. Classically, it consists of four clinical variants: Classic KS (CKS) - or Mediterranean KS-, iatrogenic KS, African KS, and AIDS-KS. All four variants are associated with Human Herpesvirus-8 (HHV-8), and they show a similar histological pattern. HHV-8 infection of endothelial cells or circulating endothelial and/or haematopoietic progenitors leads to changes in their morphology, glucose metabolism, growth rate, lifespan and gene expression, resulting in the precipitation of KS [2].

In Italy, the most commonly observed clinical variants are CKS, typically found in persons over 60 years of age, and the epidemic form, AIDS-KS, which affects younger persons with HIV infection. In HIV-positive persons, KS constitutes an AIDS-defining condition [3]. Another subvariant of KS (termed "gay Kaposi") has also been described in HIV-negative homosexuals [4] and is possibly related to the sexual transmission of HHV-8 infection [5].

The clinical onset of KS is characterised by violaceous macules and papules, which over the course of months or years tend to merge into plaques and nodules (in some cases ulcerated), which are associated with a characteristic oedema, particularly evident in the lower limbs. However, definitive diagnosis is based on histopathological evidence of spindle cell and the presence of HHV-8 latency associated nuclear antigen (LANA), in spindle cells and vascular or lymphatic endothelial cells [6].

The clinical progression of CKS is generally slow and not very aggressive, although cases with rapidly growing lesions, with signs of local invasiveness, can be observed, as well as forms that fail to respond to physical or systemic treatment. By contrast, the natural history of AIDS-KS, which can affect mucous membranes, lymph nodes, the gastrointestinal tract, and the lungs, is more aggressive, particularly in untreated HIV-infected individuals [7].

Diverse classification methods have been proposed, based on the clinical aspects and localization of lesions, which can also be assessed by roentgen-ray study, gastroscopy, and total body TC [8-10]. To define KS accurately, additional aspects can be considered, including immunological and virological parameters of HHV-8 and HIV infection, which could also be used to evaluate prognostic aspects and therapeutic indications [11-13].

Other non-invasive diagnostic techniques, in particular, telethermography and confocal microscopy, could be complementary to traditional staging instruments [14,15]. Recently, several studies have demonstrated useful applications of ultrasound in dermatology, particularly as an indicator of cutaneous fibrosis or to evaluate melanoma lesions [16,17]. Our experience suggests that skin ultrasonology, particularly when performed with an extremely high frequency probes, could be important for both the diagnosis and therapy management of KS, in association with color power Doppler flow imaging, to detect the vascular activity of the cutaneous lesions [18,19].

Over many years of ultrasound activity, we observed that skin lesions in patients with CKS were structurally more homogeneous and with a lower signal at the color power Doppler, compared to similar lesions in patients with AIDS-KS, which were less homogeneous and showed more intensive signals. Based on these observations, and after having obtained the consensus of the Ethics Committee, we conducted a randomised prospective-observational study, in which we used ultrasound to evaluate the morphology and vascularisation of erythematous-papular-angiomatous skin lesions in outpatients of the Infective Dermatology Division of the San Gallicano Institute, who we subsequently referred to the Radiology Department.

## Methods

The study population consisted of patients - with final diagnosis of KS - who presented at the San Gallicano Dermatology Institute in Rome- Italy - for the first time in 2010 and who had not been previously diagnosed or undergone to any treatment.

A total of 24 patients with a final diagnosis of KS were included in the study, of whom 16 had CKS (13 males and 4 females; median age: 70 years) and 8 had AIDS-KS (all males; median age: 47 years). All patients underwent complete clinical staging. For HIV-negative patients, we used the clinical classification criteria of Brambilla [8,13], whereas for HIV-positive patients we use a modified version of the staging of Kriegel [9] and that of Stebbing [10], based on a score from 1 to 15 (patients with a score of > 12 generally have a worse prognosis and require systemic chemotherapy, in addition to HAART). Among patients with CKS, 14 were in stage I-II-III A/B, with non-aggressive disease and slow clinical progression. The other two CKS patients were in stage IV B, showing angiomatous plaques and nodules, which were prevalently localized on the lower limbs, rapidly evolving, and associated with local complications (lymphedema and bleeding). All patients with AIDS-KS belonged to the class C, with a score of >12.

Histological examination of all of the lesions studied by ultrasound was performed on hematoxylin/eosin-stained tissue sections (4 μm) of biopsy samples, fixed in 10% buffered formaline and embedded in paraffin. Sections were also processed for immunohistochemical analysis of the expression of the endothelial associated antigens CD31, CD34 and podoplanin, a transmembrane mucoprotein described in a variety of lymphovascular neoplasms, including KS [20,21] (D2-40 MoAb, Nichirei Bioscience, Tokyo, Japan) and HHV-8 LANA (anti-HHV-8 ORF73,LNA-1, Advanced Biotechnologies Inc, USA). Testing for the immunologic condition included immunophenotyping of peripheral lymphocytes by flow cytometry. In patients with AIDS-KS, the CD3+/CD4+ lymphocyte count ranged from 125 to 1980 n/mmc (median value: 677 n/mmc). All patients were positive for HHV-8 infection, assessed by the presence of specific antibodies directed to antigens associated with the lytic and/or latent phases of infection [22]. The anti-HHV-8 antibody titers ranged from 1:80 to 1: 5120, with a median value of 1:1280. Testing for virologic parameters of HHV-8 infection was performed both on the lesion tissue and on peripheral blood. In fact, several studies have reported a correlation between HHV-8 viral load and clinical disease progression, especially for AIDS-KS [11]. The presence of HHV-8 viral genomes in plasma was evaluated and quantified using quantitative PCR (HHV-8Q real time PCR, Nanogen, Torino, Italia), with viral loads ranging from lower than 125 to 840 genome equivalents/ml). In 9 patients, viral DNA was not detectable (Table 1).

**Table 1:** Patient's characteristics and ultrasound results

Diagnosis	Age	Sex	Clinical Stage	Lesion (mm)	HHV8-DNA (copies/mL)	Ultrasound Pattern	Color-Doppler Signals
1.CKS	70	M	III A	6	652	HOMOG.	NO

2.CKS	80	M	*I A*	20	<125	HOMOG.	NO

3.CKS	56	M	I A	10	Undetectable	HOMOG.	NO

4.CKS	88	M	IV B	10	<125	HOMOG.	50%

5.CKS	70	M	II A	20	Undetectable	HOMOG.	NO

6.CKS	71	M	IV B	10	250	HOMOG.	25%

7.CKS	87	F	III A	7	520	HOMOG.	NO

8.CKS	56	F	II A	5	Undetectable	HOMOG.	NO

9.CKS	61	M	I A	6	<125	DISHOMOG.	NO

10.CKS	58	M	I A	10	Undetectable	HOMOG.	NO

11.CKS	74	M	I A	10	Undetectable	HOMOG.	NO

12.CKS	43	M	I A	<5	Undetectable	HOMOG.	NO

13.CKS	88	F	III A	7	633	HOMOG.	NO

14.CKS	56	M	III A	8	750	HOMOG.	NO

15.CKS	70	M	III A	4	450	HOMOG.	NO

16.CKS	70	M	II A	10	<125	HOMOG.	NO

17.AIDS-KS	41	M	>12	6	Undetectable	HOMOG.	NO

18.AIDS-KS	47	M	>12	4	<125	HOMOG.	25%

19.AIDS-KS	38	M	>12	4	Undetectable	CALCIF.	NO

20.AIDS-KS	59	M	>12	11	840	DISHOMOG.	50%

21.AIDS-KS	74	M	>12	9	<125	DISHOMOG.	50%

22.AIDS-KS	46	M	>12	7	230	HOMOG.	25%

23.AIDS-KS	49	M	>12	7	<125	HOMOG.	25%

24.AIDS-KS	31	M	>12	10	Undetectable	DISHOMOG.	25%

To obtain a sample that was as homogeneous as possible, we only studied those lesions with a maximum diameter between 0.4 and 2 cm and which morphologically could be defined as plaques or nodular. All patients were evaluated with ultrasound by two experts in diagnostic dermatological ultrasound (FMS and FE), under blind conditions. The images were stored on digital support and then re-evaluated in consensus by both. The ultrasound examination was performed with My-Lab 70 XVG (Esaote, Genova, Italia), using a high-frequency linear array probe (18 Mhz); for lesions with a diameter of less than 1 cm, a MyLabOne (Esaote, Genova, Italia) was also used, with a linear array probe of 22 Mhz. The settings of the devices were optimized for slow flows and superficial lesions. Written informed consent was obtained from patients. A copy of written consent is available for review by the Editor-in-chief of this journal.

## Results

**Figure 1:**
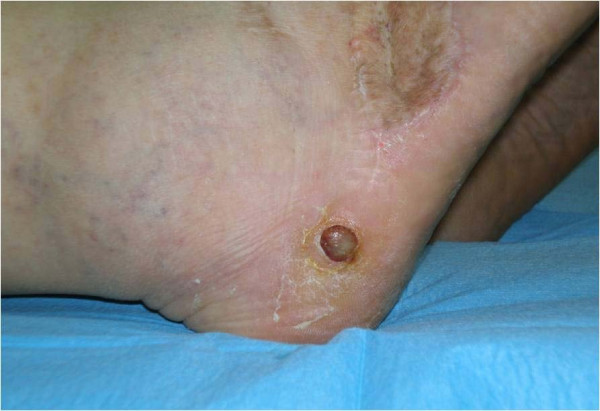
**Lesion of Classic KS**. Protruding erythemal-cyanotic nodule, with slow evolution, in a patient with Classic Kaposi Sarcoma.

**Figure 2:**
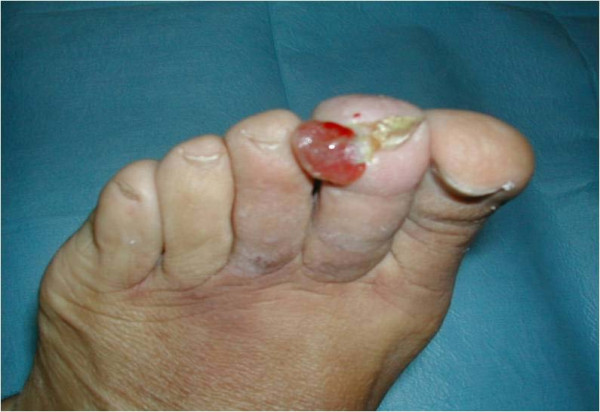
**Lesion of AIDS-KS**. Rapidly growing nodule, in a patient with AIDS-KS and severe immunodeficiency.

**Figure 3:**
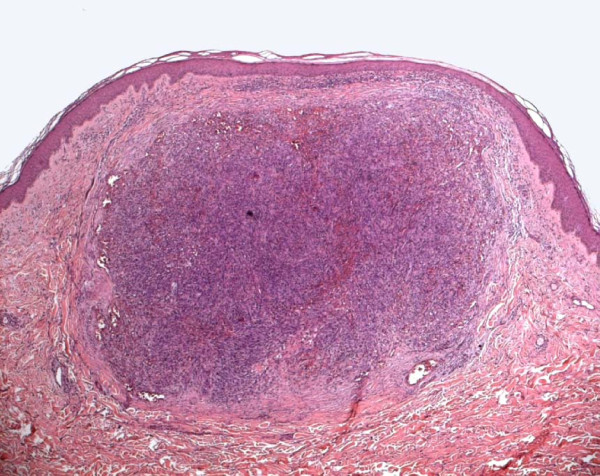
**Histology of Classic Kaposi Sarcoma (hematoxylin and eosin, 4X)**. Evident nodular proliferation of spindle cells, with hyperchromic nuclei and rare mitotic figures; presence of multiple, small, diffused and morphologically irregular vascular spaces.

**Figure 4:**
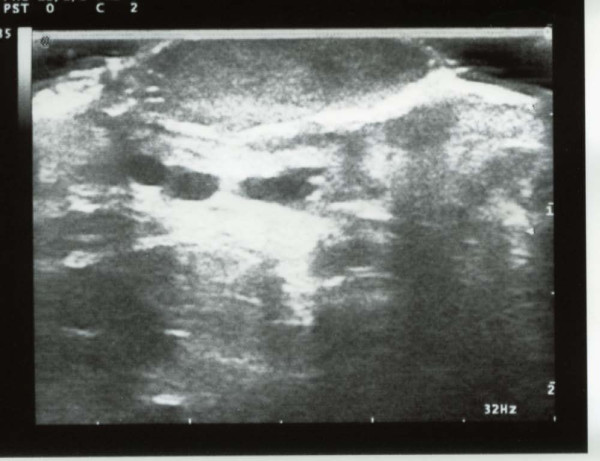
**Ultrasound image of a nodule in a patient with Classic Kaposi Sarcoma**. The formation is homogeneous, hypoechoic, with clear and well-defined contours. It involves the epidermis and derma and it is associated to ectasia of local-regional vessels in adipose sub-cutaneous tissue.

A total of 24 lesions (one per patient) were clinically observed and successively evaluated with ultrasound; of these, 16 were CKS, localised on the lower limbs (Figure 1). The lesions from the 8 patients with AIDS-KS were also localised in areas other than the lower limbs (Figure 2). All of the lesions studied by ultrasound appeared to be localized between the epidermis and the dermis, although in some cases they were also subcutaneous ( Figure 3, 4).

According to the ultrasound, in 15 of the 16 patients with CKS, the lesions, whether plaque-like or nodular, appeared to be solid and homogeneously hypoechoic, whereas in 3 of the 8 patients with AIDS-KS, the lesions were hypoechoic yet dishomogeneous (Table 1).

**Figure 5:**
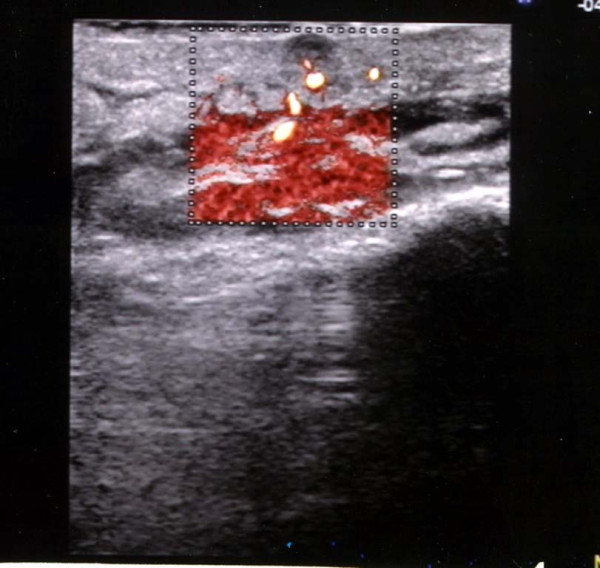
**Vascular aspects of Classic KS**. Classic Kaposi Sarcoma lesion, with slight vascularisation (only one vascular pole), in a small superficial hypoechoic lesion, is evident.

**Figure 6:**
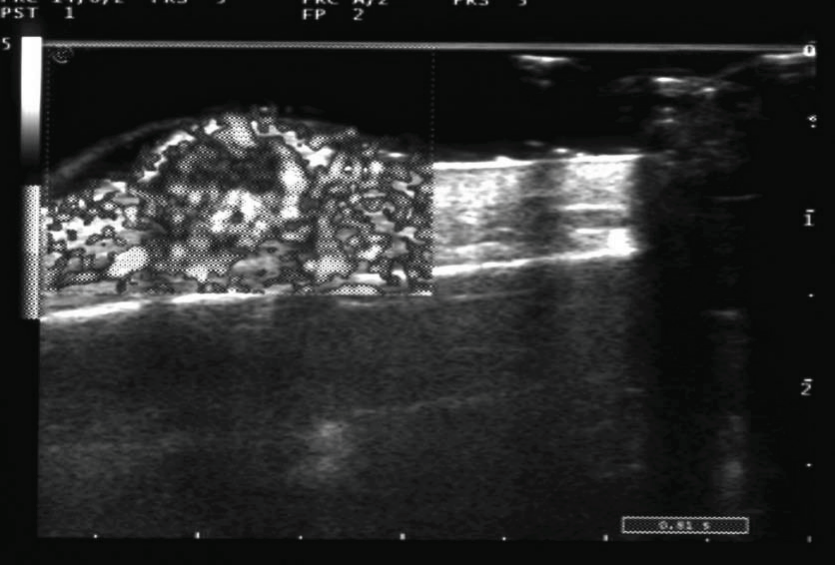
**Vascular aspects of AIDS-KS**. AIDS-KS lesion, with evident vascularisation; the monochromatic color power Doppler indicates marked vascularisation of the periphery of the nodule, with a ring-like pattern and a hypovascular central area.

According to the color power Doppler, in 6 of the 8 patients with AIDS-KS (75%), there were internal signals (Figure 5). In three of these patients, the signals were evident (Figure 6); in two of them they were present in at least 50% of the region of interest (ROI); in the remaining patient it was not possible to accurately evaluate the signal, because of the presence of considerable calcification and fibrosis. Only in 2 (16%) of the patients with CKS was there a color power Doppler signal.

According to the ultrasound, in all patients the contours of the lesions were regular, also in depth. Histologically, all of the lesions showed vascular proliferation, consisting of irregularly dilated canals, which to varying degrees were associated with bundles of spindle cells. These cells delimited irregular vascular spaces, present in the derma, at various levels, in a nodular or plaque-like state. In some patients there were telangiectasias which extended to the subcutaneous layer and which were more evident in larger lesions. An inflammatory lymphoplasmacellular infiltrate was present in all patients (Figure 3). There were no histological differences between the two KS variants.

According to the immunophenotypic analyses, all of the patients studied were positive for CD31, CD34, podoplanin and HHV8, with no differences in expression between the two variants.

## Discussion

In the literature there are few studies on ultrasound analyses of KS, and those that have been published report conflicting results. According to one study [23], the typical ultrasound pattern is a solid not homogeneous nodule, with contours that are not well-delimited and evident vascularisation according to the color power Doppler, whereas in another study [18] the lesions were reported to be hypoechoic, with a homogeneous structure and well defined contours.

Our experience is based on observations performed with very high frequency probes and a high-resolution color power Doppler, which are technologically superior to the instruments used in the past. In our study, all of the lesions were hypoechoic, with a very homogeneous structure for CKS lesions and a less homogeneous structure for AIDS-KS ones. In all cases, the contours were well defined but in many cases multi-lobulated, with good ultrasound transmission.

According to the color power Doppler, internal vascularisation was rare in CKS lesions (Table 1), whereas it was almost always present in AIDS-KS. For the AIDS-KS patients, it can be hypothesized that vascularization was related to an intense neo-angiogenesis, sustained by the HIV virus, as suggested by experimental studies [24,25]. In the two patients with CKS with a color power Doppler signal, the internal vascular signal was present in less than 25% of the ROI in one patient and in about 50% in the other. Although both patients were affected by CKS, the clinical progression was very aggressive (stage IV B), and the HHV-8 viral load was significantly higher than the mean viral load for CKS patients.

It is also possible that the relative structural homogeneity of the lesions in our study was related to the small size of most lesions and that the structural dishomogeneity was actually produced by phenomena such as fibrosis and intra-neoplastic degeneration with areas of necrosis, which is typical of larger neoplasia, in which the blood intake becomes in some way inadequate. This is evident in Figure 6, where the central areas of tumor lesion are clearly hypovascular, in the presence of a rich peripheral vascular ring; however, this observation should need to be confirmed by studies on larger number of subjects. The finding that the contours of the lesions were regular, even deep down, is instead surprising for the aggressive forms of AIDS-KS; nonetheless, this could be attributable to the relatively small size of the lesions, which were perhaps observed in an initial pre-infiltrative phase of the disease.

## Conclusions

Although the ultrasonography of KS lesions is not pathognomonic (similar findings can been found in other flogistic and non-flogistic pathologies), we can conclude that it allows clinically similar pathologies (such as angiomas and artero-venous malformations in the growth phase) to be excluded. Moreover, the ultrasound pattern observed in this study differs from that reported in previous studies. Although we evaluated a limited number of patients in a single clinical centre, our results show that small CKS lesions are relatively uniform, superficially, hypo echoic, and with well defined contours; they are usually located between the epidermis and the dermis and lack color power doppler signals in the less aggressive forms, whereas vascularisation is evident in the rapidly evolving forms.

In patients with AIDS-KS, the ultrasound pattern in B-mode was similar to that for the other group, although, according to the color power Doppler, the lesions were all hypervascular. This finding is consistent with the presence of marked neoangiogenesis in the HIV-related variants, which is closely related to the activity of the HIV-1 virus on the endothelial cells [24,25]. However, we cannot draw definitive conclusions regarding the prognostic significance of hyper vascularisation in this group, given the brevity of the follow-up for these patients and the immediate starting of antiretroviral therapy.

Thus in our opinion, in patients with CKS, ultrasound evaluation of lesions with the color power Doppler study could be used as a non-invasive diagnostic technique for distinguishing between forms with rapid clinical progression - thus requiring therapy - and less aggressive forms, requiring only follow-up. Although this proposal needs to be evaluated with additional studies, including larger number of patients, given its low cost and non-invasiveness, this technique could be immediately used, at least in experienced centres, and included in the diagnostic-therapeutic course for KS.

## Competing interests

The authors declare that they have no competing interests.

## Authors' contributions

FMS conceived of the study and participated in its design and coordination. AL made the clinical diagnosis and the follow up of patients. FE performed the ultrasound and color Doppler analysis.

PCF carried out the immunological and virological determinations. CC performed the histological diagnosis. ADC coordinated the study. All authors read and approved the final manuscript
